# An Integrated biomarker approach for explaining the potency of exogenous glucose on transportation induced stress in *Labeo rohita* fingerlings

**DOI:** 10.1038/s41598-021-85311-5

**Published:** 2021-03-11

**Authors:** Abhilipsa Biswal, Prem Prakash Srivastava, Gopal Krishna, Tapas Paul, Prasenjit Pal, Subodh Gupta, Tincy Varghese, Manish Jayant

**Affiliations:** 1 ICAR-Central Institute of Fisheries Education, Mumbai, 400 061 India; 2 College of Fisheries, Central Agricultural University, Lembucherra, Tripura 799210 India

**Keywords:** Biochemistry, Biological techniques, Physiology

## Abstract

Transportation of fish seed is a complex phenomenon associated with multiple kinds of stressors that simultaneously affect the fish in a confined environment, causing stress and mortality. The present study investigated the stress-relieving effect of exogenous glucose as a water additive in different concentrations (0.1, 0.2, 0.3, and 0.4%) during simulated transportation (12 h) of *L. rohita* fingerlings. The integrated biomarker response (IBR) index is a holistic tool to determine the optimum dose of exogenous glucose for mitigating transportation stress in fish. Based on selected biomarkers related to the stress hormone, serum biochemistry, oxidative stress, and HSP70 mRNA expression, the IBR index is calculated for each treatment and control group. The result showed a significant change in the level of stress hormone cortisol, enzymes (SGPT, LDH, MDH, SOD, CAT) and metabolites (serum glucose, triglyceride, creatinine) along with an upregulation in liver HSP70 mRNA expression. IBR index suggests that 0.2% glucose exhibited the lowest multi-biomarker stress response in comparison to other treatments and control. Therefore, the use of 0.2% glucose as a water additive will provide a solution to transportation induced stress in *L. rohita* fingerling and will underwrite the success of grow-out fish culture in days to come.

## Introduction

With an extensive growth rate of population, it has been predicted that by the end of 2050, there will be 9.7 billion inhabitants in the world, and it is creating a global concern for the overall nutritional safety^1,2^. Fish is contemplated as an excellent animal protein source, and with 80 million tons (USD 231.6 billion) of production, aquaculture is regarded as a significant contributor to the food and nutritional security of the world population^3^. However, several constraints impede the growth and sustainability of the aquaculture sector, and transportation stress is one of the significant concerns among them^4^. Transportation of fish fingerlings leads to a higher rate of energy depletion and eventually mortality due to interactive involvement of different types of a stressor such as handling, netting, grading, packing, vibration, water movement, deterioration of physicochemical parameter of water acting concurrently on fish for a certain period in a confined environment^5,6^. It has been reported that transportation stress can cause high mortality, which can be counted as high as 90% of the total transported fish^7^.

The freshwater aquaculture system in India is primarily dominated by carps and contributes 87% to total aquaculture production^8^. *L. rohita* occupies a principal position due to its high commercial value, acceptability to artificial diet, higher growth rate, the texture of meat, and consumer preference^9^. But the major concern associated with this species is its higher degree of vulnerability towards confinement and transportation stress, which makes it a suitable species for the present study^10^.

The use of an integrated biomarker response (IBR) index provides a holistic approach to assess the level of stress in fish^11^. During transportation, changes at the biochemical, cellular, and physiological levels of *L. rohita* create a metabolic upheaval at cellular and molecular levels^12^. Enzymes and hormones are used as a biomarker for assessing the level of stress in fish since they act as the first line of indicators of transportation stress^11,13^. Stress hormone such as cortisol act as primary stress biomarker due to the activation of the hypothalamus-pituitary-chromaffin axis and the hypothalamus-pituitary-interrenal axis during long term transportation, which results in the release of catecholamine and cortisol respectively^8,14^. Oxidative stress enzymes (SOD, CAT) and Metabolizing enzymes (GPT, LDH, and MDH) acts as secondary stress biomarkers during the transportation of fish^12,15^. Further, change in serum triglyceride and creatinine levels in fish to cope with additional energy demand due to transportation stress act as potential biomarkers^8,16^. Furthermore, under transportation stress conditions, HSP 70 mRNA expression is a reliable marker of cellular stress in fish^16,17^. Therefore, IBR can be used as a reliable approach to determine the optimum dose of water additive by measuring the level of stress biomarkers during transportation.

At present, several water additives are being used for the transportation of fish. Recently, the use of anesthesia for mitigating transportation stress has gained attention. The use of several salt and anesthesia during transportation has reported alleviating oxidative stress and hydromineral imbalance in transported fishes^4,18,19^. However, the use of salt during transportation has shown no reduction in stress response and osmoregulatory disturbance in some fishes^18^. Further, few researchers have reported that anesthetics can further increase oxidative stress and improper ammonia excretion^20^.

On the note, due to the elevated osmoregulatory disturbances during transportation, the energy demand of the fish increases to maintain the ionic balance of the body, which needs to be compensated by the use of exogenous organic energy fuels^21^. Glucose is a carbohydrate having molecular formula of C_6_H_12_O_6_ and is considered the most important energy source in the energy metabolism of vertebrates^22^. During stress, glycolysis and gluconeogenesis occur in the fish body to provide glucose and energy^12^. So, the idea behind addition of glucose as an exogenous energy source is to sustain the excess metabolic energy demand during stress conditions. In general, it is observed that carps are more tolerant to glucose than carnivorous fishes. However, baseline information on the use of exogenous glucose as a water additive for mitigating transportation stress in fish is still not reported. Therefore, the present study aimed to standardize the most efficient dose of exogenous glucose for long-term transportation of *L. rohita* based on selected biomarkers.

## Results

### Water quality biomarkers

A significant (p < 0.05) higher total ammonia–nitrogen was observed in control as compared to treatments after 12 h transportation. Further, the lowest total ammonia–nitrogen concentration was observed in T_2_ (0.2% glucose) in comparison to other treatments (Supplementary Table S1). On the contrary, the pH of the control group was found to be substantially (p < 0.05) lesser in comparison to the treatments after transportation (Supplementary Table S1).

### Serum biomarkers

**Figure 1:**
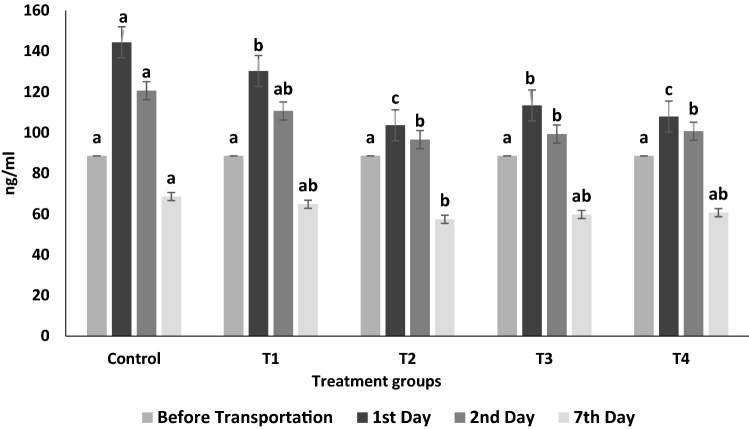
Serum cortisol concentration in *Labeo rohita* fingerlings before and after transportation (mixed with glucose in ambient water). Data is represented in mean ± SE, p value ≤ 0.05 and various superscripts represent the significant levels.

The serum cortisol hormone level was significantly (p < 0.05) higher in control fish as compared to treatments on 1st and 2nd day sampling (Fig. 1). Among the treatments, the lowest cortisol level was observed in 0.2% glucose group on both 1st day and 2nd day sampling. At 7th day sampling, serum cortisol levels of all the treatments and control were insignificantly different from each other.

The SGPT activity was significantly (p < 0.05) lower in all the treatment groups compared to control on the 1st and 2nd day sampling. The least SGPT activity was observed in the T_2_ (0.2%) group in comparison to the other treatments and further, T_3_ (0.3%) andT_4_ (0.4%) were found to be insignificant to each other throughout the experimental period (Table 1).

**Table 1:** SGPT, Serum creatinine, Serum triglyceride and Serum glucose concentration in *Labeo rohita* fingerlings before and after transportation (mixed with glucose in ambient water).

Parameter	Treatments	Before transportation	1st day	2nd day	7th DAY
SGPT (IU L^−1^)	T_0_ (Control)	18.12^c^ ± 1.12	36.24^a^ ± 1.73	24.46^a^ ± 1.39	21.55^a^ ± 1.29
	T_1_ (0.1%)		29.91^b^ ± 1.42	22.57^a^ ± 1.06	20.25^b^ ± 1.13
	T_2_ (0.2%)		23.92^c^ ± 0.94	21.29^b^ ± 1.14	18.75^c^ ± 0.92
	T_3_ (0.3%)		24.92^c^ ± 1.43	20.61^b^ ± 1.05	19.41^bc^ ± 1.21
	T_4_ (0.4%)		23.30^c^ ± 1.67	20.24^b^ ± 1.70	19.75^bc^ ± 1.40
Serum creatinine (mg dL^−1^)	T_0_ (Control)	0.30^a^ ± 0.01	1.06^a^ ± 0.04	0.66^a^ ± 0.03	0.41^a^ ± 0.03
	T_1_ (0.1%)		0.81^b^ ± 0.04	0.52^b^ ± 0.02	0.36^ab^ ± 0.02
	T_2_ (0.2%)		0.65^bc^ ± 0.05	0.40^c^ ± 0.03	0.30^b^ ± 0.01
	T_3_ (0.3%)		0.67^bc^ ± 0.06	0.39^c^ ± 0.02	0.31^b^ ± 0.02
	T_4_ (0.4%)		0.64^c^ ± 0.06	0.40^c^ ± 0.03	0.33^ab^ ± 0.01
Serum Triglyceride (mg dL^−1^)	T_0_ (Control)	131.35^a^ ± 5.29	254.96^a^ ± 8.77	201.60^a^ ± 9.51	143.74^a^ ± 4.39
	T_1_ (0.1%)		211.44^b^ ± 9.55	177.59^b^ ± 7.91	140.27^a^ ± 5.77
	T_2_ (0.2%)		176.73^c^ ± 6.36	151.66^c^ ± 6.51	132.95^a^ ± 6.65
	T_3_ (0.3%)		177.57^c^ ± 7.50	151.88^c^ ± 9.56	134.95^a^ ± 5.94
	T_4_ (0.4%)		178.06^c^ ± 7.31	154.36^c^ ± 9.23	134.16^a^ ± 6.20
Serum glucose (mg dL^−1^)	T_0_ (Control)	39.17^a^ ± 2.34	78.56^a^ ± 5.76	68.59^a^ ± 4.97	41.64^a^ ± 2.23
	T_1_ (0.1%)		67.22^b^ ± 3.35	52.25^b^ ± 3.54	37.83^b^ ± 2.19
	T_2_ (0.2%)		48.94^d^ ± 3.46	42.13^d^ ± 2.05	36.54^b^ ± 1.87
	T_3_ (0.3%)		52.87^d^ ± 3.01	47.59^c^ ± 3.12	38.2^b^ ± 1.73
	T_4_ (0.4%)		62.23^c^ ± 2.98	50.18^c^ ± 2.84	39.71^ab^ ± 1.27

Data is represented in mean ± SE, p value ≤ 0.05 and various superscripts represent the significant levels.

Serum triglyceride level and creatinine activity were significantly (p < 0.05) higher in control groups as compared to treatments on 1st and 2nd sampling days (Table 1). Among the treatments, the lowest serum triglyceride level and creatinine activity was found in T_2_ (0.2%) during the experimental period.

Serum glucose level was substantially (p < 0.05) higher in control as compared to treatments on all sampling days (Table 1). There was no significant difference among T_2_ (0.2%)_,_ T_3_ (0.3%) and T_4_ (0.4%) on 7th day sampling with lowest serum glucose level in T_2_ group.

### Tissue biomarkers

A significantly (p < 0.05) higher LDH and MDH activity were observed in liver and muscle of control group as compared to treatments after transportation (Supplementary Table S2). Among all the treatments, the 0.2% glucose group exhibited the lowest LDH and MDH activity in both liver and muscle tissue in all the sampling days.

SOD and CAT activity were substantially (p < 0.05) higher in the liver and gill tissues of control groups in comparison to treatments on the 1st day of sampling post-transportation (Supplementary Table S3). The lowest elevation of SOD and CAT activity was observed in the T_2_ (0.2%) group between the treatments. However, T_3_ (0.3%) and T_4_ (0.4%) were found to be insignificant to each other throughout the experimental period (Supplementary Table S3).

G-6 phosphatase activity was significantly higher in the liver of control groups than treatments during the experimental period (Supplementary Fig. S1). Further, the lowest activity was observed in 0.2% exogenous glucose treatment on 1st and 2nd day of sampling.

### mRNA expression

**Figure 2:**
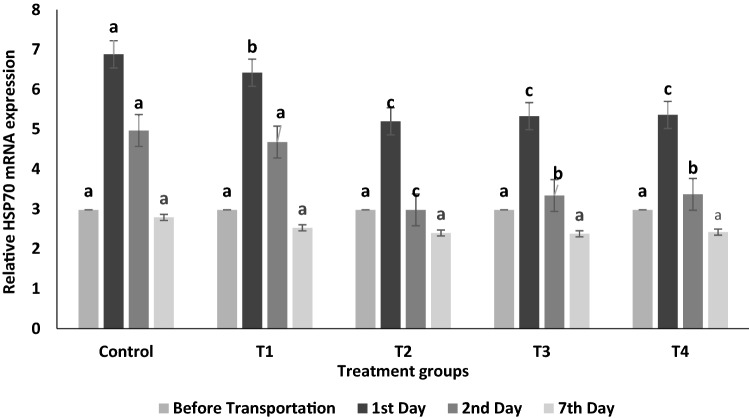
HSP70 mRNA expression in liver of *Labeo rohita* fingerlings before and after transportation (mixed with glucose in ambient water). Data is represented in mean ± SE, p value ≤ 0.05 and various superscripts represent the significant levels.

After simulated transportation of 12 h, a significant increase in HSP70 mRNA expressions was found in the liver of control fishes (T_0_) in comparison to the treated fishes (P < 0.05; Fig. 2). Further, 0.2% exogenous glucose (T_2_) showed the lowest upregulation of HSP70 mRNA expressions as compared to other treatments.

### Integrated biomarker response (IBR)

**Figure 3:**
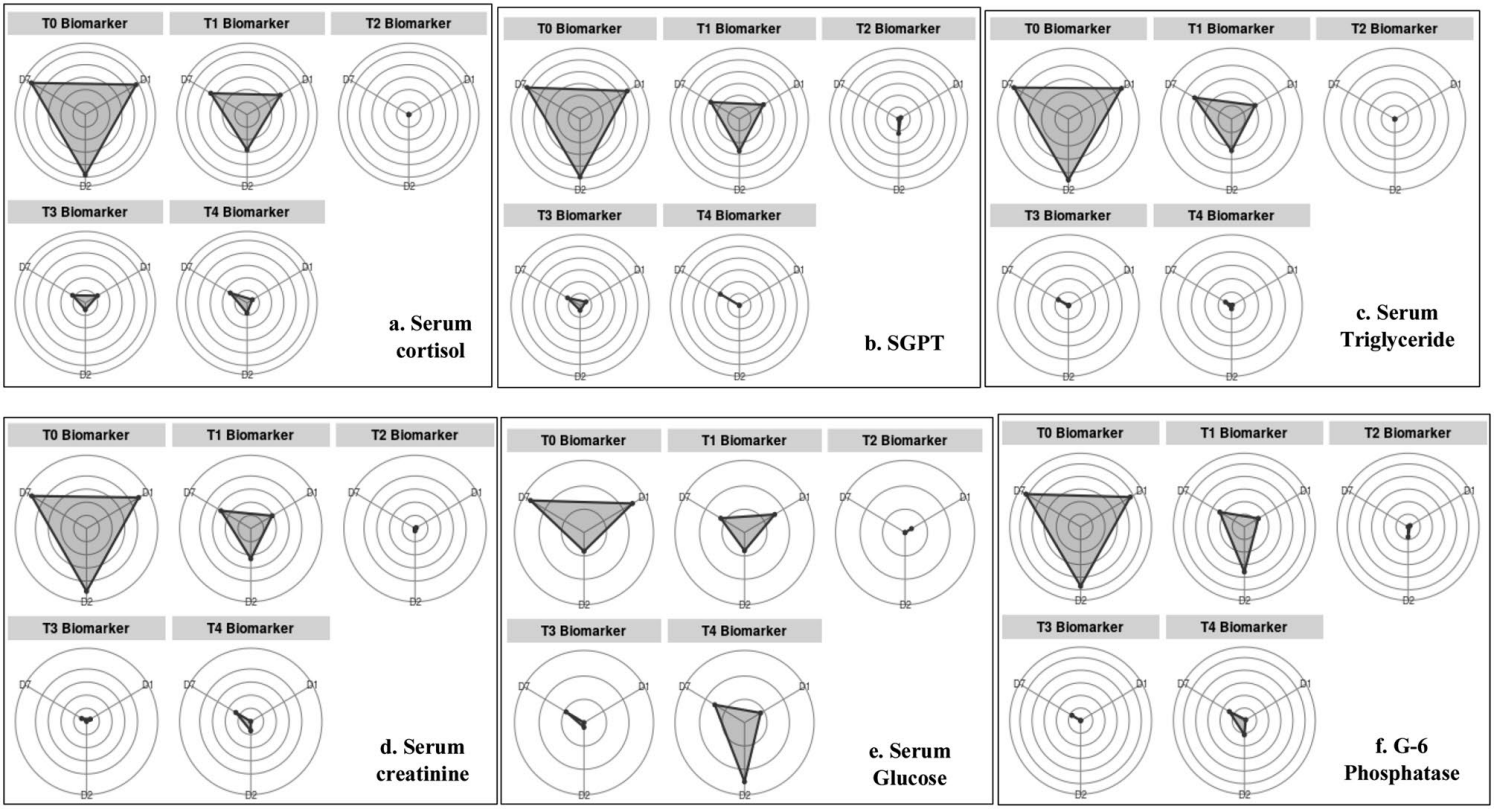
IBRstarplots of different biomarkers (**a**) Serum cortisol, (**b**) SGPT, (**c**) Serum Triglyceride, (**d**) Serum creatinine, (**e**) Serum Glucose, (**f**) G-6 Phosphatase in liver tissue in each treatment of glucose (T_1_: 0.1%, T_2_: 0.2%, T_3_: 0.3% and T_4_: 0.4%) and control.

**Figure 4:**
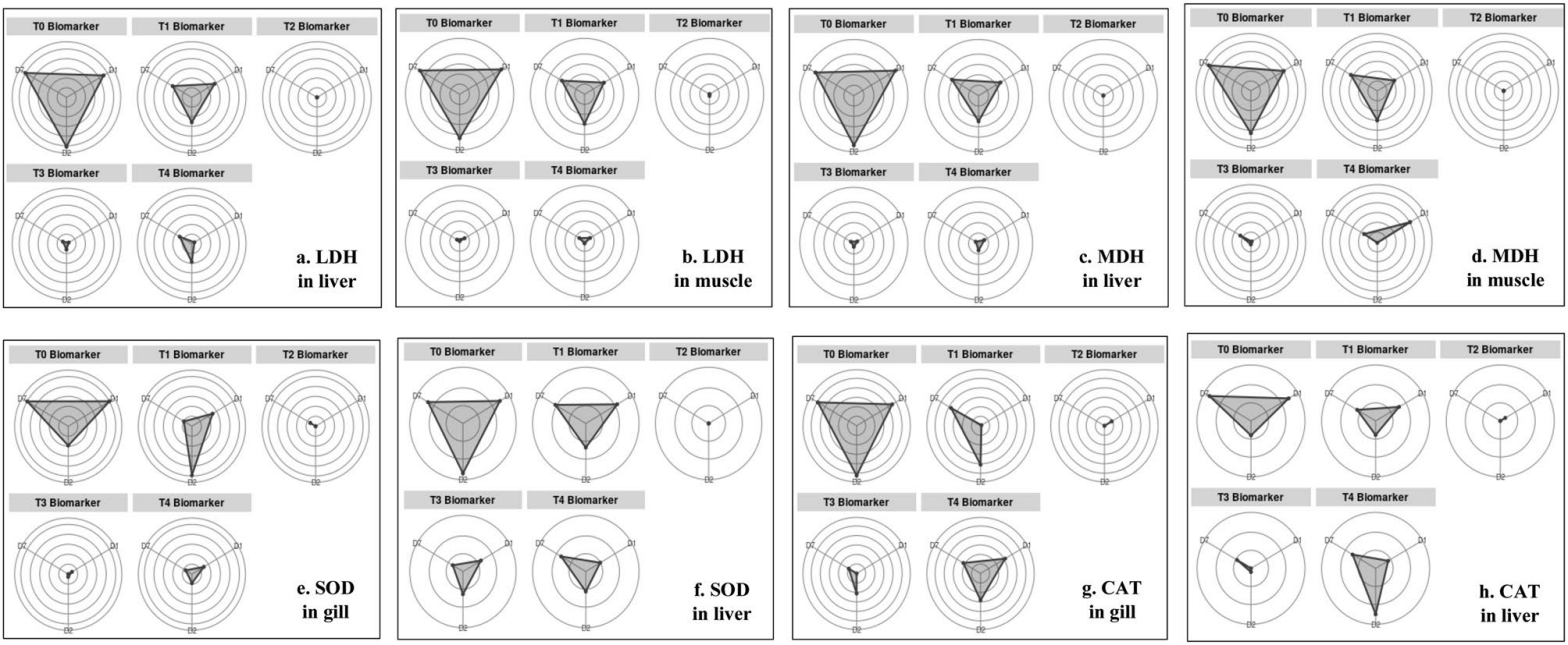
IBR star plots for multi-biomarker responses such as (**a**, **b**) LDH, (**c**, **d**) MDH activity in liver and muscle and (**e**, **f**) SOD and (**g**, **h**) CAT activity in gill and liver tissue at each treatment of glucose (T_1_: 0.1%, T_2_: 0.2%, T_3_: 0.3% and T_4_: 0.4%) and control (T_0_) in *L. rohita*.

IBR is a potential tool for evaluating the optimum dose of glucose as a water additive by using the multi-biomarker response. Stress hormone (Cortisol), Oxidative stress (SOD, CAT), Metabolizing enzymes (SGPT, LDH, and MDH) and serum biomarkers (triglyceride, creatinine, and glucose) activity of *L. rohita* exposed to long term transportation stress were standardized and data are presented in star plots (Figs. 3, 4). The IBR index showed a differential response of biomarkers in each treatment of glucose and control. Serum cortisol showed the highest IBR value in control (7.79 ± 0.14), while the lowest value was observed in 0.2% glucose (T_2_) group (Table 2). Among the oxidative stress biomarkers, the highest IBR index was observed for control (SOD: 4.82 ± 0.08; 8.63 ± 0.15, CAT: 7.8 ± 0. 11; 4.8 ± 0.05 in gill and liver tissue), and the lowest IBR value was found in 0.2% exogenous glucose (T_2_) group for both SOD and CAT (Fig. 4; Table 2). In metabolizing enzymes, IBR index showed a similar trend with highest values for control (LDH: 7.79 ± 0.10; 7.08 ± 0.18 and MDH: 7.11 ± 0.21; 6.59 ± 0.26 for liver and muscle) followed by 0.1% glucose group (LDH: 2.14 ± 0.07; 2.25 ± 0.05 and MDH: 2.35 ± 0.04; 2.48 ± 0.09 for liver and muscle) while the least IBR index was found in 0.2% glucose treatment (Fig. 4; Table 2). Interestingly, in G-6 phosphatase biomarker in liver tissue, the lowest IBR value was observed for T_3_ (0.3%) treatment (Table 2). Among the serum biomarkers such as GPT, triglyceride, creatinine, and glucose minimum area of star plot was covered in T_2_ (0.2%), signifying the lowest IBR index due to transportation stress (Fig. 3; Table 2). Therefore, T_2_ treatment (0.2% glucose) showed the lowest IBR values of different biomarkers compared to other treatments and control, signifying the highest efficacy in mitigating stress during transportation.

**Table 2:** Summary of IBR mean values of different biomarkers in each treatment of glucose and control. Data is represented in mean ± SE, p value ≤ 0.05.

Biomarkers		Treatment groups				
		T_0_ (control)	T_1_ (0.1%)	T_2_ (0.2%)	T_3_ (0.3%)	T_4_ (0.4%)
Serum biomarkers	Cortisol (ng mL^−1^)	7.79 ± 0.14	3.17 ± 0.04	00.00	0.29 ± 0.002	0.27 ± 0.001
	GPT(IU L^−1^)	8.1 ± 0.07	2.3 ± 0.02	0.03 ± 0.002	0.17 ± 0.005	00.00
	Triglyceride(mg dL^−1^)	6.88 ± 0.04	2.03 ± 0.01	00.00	0.01 ± 0.001	0.02 ± 0.001
	Creatinine(mg dL^−1^)	7.54 ± 0.02	1.72 ± 0.03	00.00	0.02 ± 0.003	0.1 ± 0.002
	Glucose(mg dL^−1^)	4.8 ± 0.03	1.76 ± 0.01	00.00	0.08 ± 0.004	3.14 ± 0.08
Oxidative stress biomarkers	SOD liver (U mg protein^−1^)	8.63 ± 0.15	4.44 ± 0.05	00.00	1.44 ± 0.05	2.04 ± 0.02
	SOD gill (U mg protein^−1^)	4.82 ± 0.08	2.13 ± 0.03	00.00	0.01 ± 0.001	0.34 ± 0.007
	CAT liver (mmole mg protein^−1^ min^−1^)	4.8 ± 0.05	1.76 ± 0.08	00.00	0.08 ± 0.002	3.14 ± 0.05
	CAT gill (mmole mg protein^−1^ min^−1^)	7.8 ± 0. 11	1.77 ± 0.02	00.00	0.23 ± 0.001	2.37 ± 0.03
Metabolizing biomarkers	LDH liver (micromoles mg protein^−1^ min^−1^)	7.79 ± 0.10	2.14 ± 0.07	00.00	0.06 ± 0.003	0.42 ± 0.005
	LDH muscle (micromoles mg protein^−1^ min^−1^)	7.08 ± 0.18	2.25 ± 0.05	00.00	0.02 ± 0.001	0.07 ± 0.003
	MDH liver (micromoles mg protein^−1^ min^−1^)	7.11 ± 0.21	2.35 ± 0.04	00.00	0.04 ± 0.003	0.1 ± 0.002
	MDH muscle (micromoles mg protein^−1^ min^−1^)	6.59 ± 0.26	2.48 ± 0.09	00.00	0.05 ± 0.002	0.76 ± 0.008
	G-6 phosphatase (nanomoles)	7.71 ± 0.12	1.76 ± 0.09	0.02 ± 0.001	00.00	0.21 ± 0.001

### Survival

**Figure 5:**
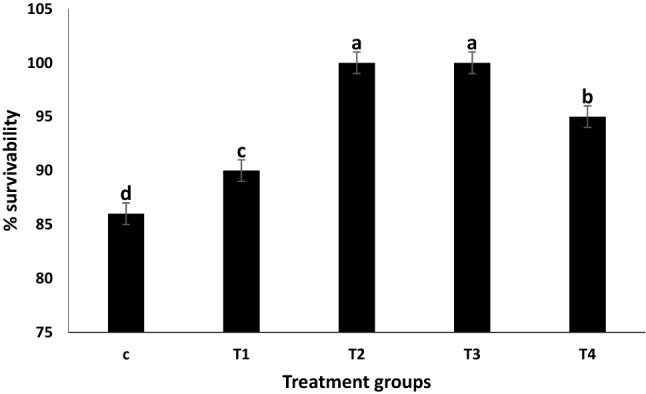
Cumulative Percentage survivability of *Labeo rohita* fingerlings during and after transportation (mixed with glucose in ambient water). Data is represented in mean ± SE, p value ≤ 0.05 and various superscripts represent the significant levels.

A significantly higher cumulative survivability was recorded in all the treatment groups compared to control after seven days of post transportation. Further, among the treatments highest survivability was found in T_2_ and T_3_ groups (0.2% and 0.3% glucose, respectively) (Fig. 5).

## Discussion

A significantly higher total ammonia–nitrogen was observed in control as compared to treatments after 12 h transportation, which might be a consequence of nitrogenous waste excretion due to the higher metabolic rate of fish during transportation^8,23^. Alteration of water pH during transportation is due to integrated result of two different reactions such as conversion of ammonia to ammonium ion and CO_2_ to carbonic acid. The formation of ammonium ion is an alkaline reaction that might have increased the pH of all experimental groups. Interestingly, a substantial decrease of water pH was observed in control and 0.1% exogenous glucose group after transportation. It might be attributed to long term transportation stress, which has caused higher CO_2_ excretion because of increased ventricular motion and respiration rate^24^. Thus, conversion of CO_2_ to carbonic acid made transport water acidic, resulting in decreased pH in the control and T_1_ (0.1%) group (Supplementary Table S1). It also signifies higher transportation stress in control and 0.1% exogenous glucose group compared to other treatments.

Serum Cortisol level is contemplated as the most potent stress biomarker in fish^25^. In the present study, the cortisol hormone level was substantially higher in control fish as compared to treatments on 1st and 2nd day sampling (Fig. 1). The results of the present study are in agreement with Mirghaed et al*.*^14^, who found a significant elevation in serum cortisol level of transported *Cyprinus carpio* in comparison to non-transported fish. Transportation stress causes ionic imbalance and osmoregulatory disturbance in *Oncorhynchus kisutch*, *Salmo gairdneri*, and *Micropterus dolomieu* juveniles, thus maintaining the osmoregulatory balance, serum cortisol increases all the other metabolic activity and aid in energy mobilization during stress^26,27^. Further among the treatments, the lowest cortisol level was observed in the 0.2% exogenous glucose group, which might be due to lower energy demand by the fish to sustain all other metabolic activity for energy mobilization during the experimental period^28^.

SGPT can be accounted as a dependable biomarker for indicating hepatic damage of fish during stressful conditions^29^. The probable mechanism of higher SGPT in control fish may be due to increased mobilization of amino acid for energy production via transamination to produce ATPs by the Krebs cycle in the liver^30,31^. When the hepatocyte cells are damaged, the transaminase enzyme leaks out into the bloodstream, and as a result, SGPT activity increased^12^. Similarly, Pakhira et al.^8^ reported a rise of the SGPT level in *L. rohita* subjected to transportation stress. Further, 0.2% glucose (T_2_) showed the lowest SGPT activity due to the lesser need for amino acid mobilization for sustaining the energy demand as most of the energy is provided by the exogenous source of glucose.

Rise in serum triglyceride level in control fish may be attributed to cortisol hormone-induced lipolytic action leading to increased production of triglycerides in the fish body, which further acts as a substrate for gluconeogenesis, later glycolysis, and energy generation^32^. A similar report on the increase of serum triglyceride levels was observed in channel catfish under transportation stress^16^. The lowest triglyceride level was observed in treatment with 0.2% exogenous glucose which indicates the efficacy of enabling the fish to lessen the additional energy demand during long term transportation.

Creatinine is formed from the breakdown of creatine phosphate in muscle tissue during stress and acts as a biomarker of kidney damage and impaired renal function in fish. Transportation stress leads to an increase in the serum creatinine level of control fish compared to all the treatments, indicating the weakened kidney function of transported control fish. Pakhira et al.^8^ reported a similar increase in serum creatinine level of *L. rohita* transported at different densities.

Serum glucose can be considered as a reliable biomarker of the secondary stress responses in fish^16^. In the present study, a significant elevation of serum glucose level along with higher G-6 phosphatase activity was observed in control and T_1_ (0.1% glucose) groups which might be attributed to higher transportation stress leading to energy depletion and further gluconeogenesis to produce endogenous serum glucose (Table 1). Further, serum glucose level was significantly high in 0.3 and 0.4% exogenous glucose groups despite lower G-6 phosphatase activity indicating a higher rate of exogenous glucose intake by the fish resulting in hyperglycemia condition^33^. Similar results of the increase of serum glucose were observed in *C. carpio* during transportation stress^14^.

Carbohydrate metabolizing enzymes are considered as potential stress indicators in fish^34,35^. Due to transportation stress, glycogen levels get exhausted, so LDH enzymes help in converting lactate into glucose and thus aid in gluconeogenesis for further energy generation^36^. It results in higher LDH activity of control fish during the 1st and 2nd sampling post-transportation (Supplementary Table S2). The results of the present study are in agreement with Chatterjee et al.^12^ who found a significant increase in LDH activity of *L. rohita* due to confinement stress. Rise in MDH activity in control fish due to transportation stress might be attributed to increased amino acid mobilization, so the oxaloacetate being a product of amino acid metabolism, takes part in gluconeogenesis by the activity of MDH enzyme^36^. Similarly, an increase in MDH activity was observed in *O. niloticus* exposed to confinement stress^33^. Due to the higher potency of 0.2% glucose treatment, fish has lesser demand for additional energy during the long-term transportation stress resulting in the lowest LDH and MDH activity.

Variation in antioxidant enzymes activity can be used as the indicator of stress in fish^11,37^. Oxidative stress biomarker such as SOD catalyzes the conversion of the superoxide anion into hydrogen peroxide and molecular oxygen, invariably CAT activity decomposes the hydrogen peroxide into oxygen and water^38^. SOD and CAT activity were significantly (P < 0.05) higher in the liver and gill tissues of control groups in comparison to all the treatments on the 1st day of sampling post-transportation (Supplementary Table S3). This finding concurs with the earlier observation where transportation of channel catfish led to an increase of CAT and GSH-PX^16^. The probable mechanism of higher SOD and CAT activity may be due to the upregulated synthesis of ROS in the control group, indicating an increase in the metabolism of fish due to a higher degree of transportation stress^15^. However, the lowest elevation of oxidative stress activity was observed in the T_2_ treatment simplifying the efficacy 0.2% exogenous glucose in providing the additional energy source for the lower rate of metabolic activity leading to lesser ROS generation.

During stress conditions in fish, glucose is produced from non-carbohydrate sources by gluconeogenesis under the influence of cortisol^33^. Both Glucose-6 phosphatase and Fructose 1,6-bisphosphatase are the important enzymes of gluconeogenic pathway. However, glucose-6 phosphatase completes the final step reaction in gluconeogenesis pathway and thus, it plays a vital role in homeostatic regulation of blood glucose level. Therefore, the final step enzyme glucose-6-phosphatase was targeted, for concluding about the occurrence of gluconeogenesis during transportation stress. G-6 phosphatase activity was elevated significantly in the liver of control fishes compared to treatments during the experimental period (Supplementary Fig. S1). A similar increase of G-6 phosphatase activity was observed in *L. rohita* in higher packing density and increased transportation period^12^.

HSP 70 is considered as a potential biomarker of cellular stress since it protects the immature polypeptides and controls the correction of protein misfolding during stress in fish^17,39^. A significant (P < 0.05) upregulation of liver HSP70 mRNA expressions was observed in control fish in comparison to treatments after 12 h simulated transportation (Fig. 2)*.* Similarly, transportation stress leads to a significant elevation of HSP70 mRNA expression in *Ictalurus punctatus*^16^. Further, the use of exogenous glucose (0.2%) showed the lowest relative mRNA expression indicating its efficacy for relieving the stress of transported fish.

IBR index showed that the addition of 0.2% exogenous glucose in transported water results in the lowest stress response, signifying it as the optimum dose for mitigating transportation stress in *L. rohita* (Table 2). In agreement with IBR findings, T_2_ (0.2% glucose) treatment exhibited the lowest alteration in biochemical profiles, oxidative stress, hepatic HSP70 mRNA expression, and higher cumulative survival in *L. rohita* on all sampling days (Figs. 1, 2, 5; Table 1).The IBR index showed higher mean value in the control group for each biomarker, attributing to increased oxidative stress, alteration in hormones, and metabolizing enzymes to meet energy demand during transportation stress^8,14^. Similar studies of measuring stress response by IBR were reported by Li et al. and Paul et al*.* in different organs of fish exposed to pollutant stress^11,40^.

In the present study, lower survivability of the control group may be due to continuous exposure to stress, which eventually leads to energy depletion and death of the fish (Fig. 5). The probable reason for higher survivability in 0.2 and 0.3% glucose treatment might be attributed to efficient energy supplementation by the exogenous source of glucose, which has helped to decrease the metabolic activity of the body. On note, Presence of Glut -1, a transmembrane transporter protein, has been reported in the gill area of fishes^41,42^. Further, it has been reported that stress induce release of catecholamine and increased ventilation rate of gill^43^, leading to extrication of body ionic content (sodium, chloride) into environment water, causing osmoregulatory disturbance. So, here it is hypothesized that although the fresh water fish rohu doesn’t drink water, the presence of exogenous glucose in the environmental water might have led to the facilitated diffusion of glucose along with the lost ions across the plasma membrane of gill through Glut transporter. Hence, the fulfilment of excess energy demand in stressed fish might have been attributed to the role of glucose as an important source of energy in fishes.

In conclusion**,** the study establishes that enzymes, hormone, and HSP70 mRNA expression can be successfully deployed as biomarkers to evaluate the degree of transportation stress in carps. The study of glucose-6-phosphatase enzyme activity indicated glucose homeostasis in the transported fish; however, for a better understanding of glucose metabolism glucokinese/hexokinase enzyme activity can also be studied in future research. Further, 0.2% exogenous glucose treatment is the lowest dose, which showed the highest cumulative survival of *L. rohita* fingerlings. So, long term transportation of *L. rohita* fingerlings with 0.2% exogenous glucose solution will provide best quality of transported seed and reduce a significant amount of fish mortality. The findings of the present study can be widely applicable to all other carp fish species being transported for a long distance up to culture ponds.

## Methods

### Experimental animal

*L. rohita* fingerlings (4.5 ± 0.5 g) (mean weight ± Standard deviation) were commercially procured from Pen, Raigadh district of Maharashtra, India (18.7127°N, 73.0272°E). The procured fishes were treated with mild salt dip treatment (1% NaCl solution for 30 s) followed by potassium permanganate (KMnO_4_) dip treatment (2 ppm for 2 min) following the method of Bera et al.^44^. The treated fishes were transferred to five circular fiberglass reinforced plastic tank (1000 L capacity) to acclimatize in laboratory conditions for three weeks. The fishes were maintained with continuous aeration, fed @ 3% of their body weight daily twice a day and excess feed was removed by siphoning daily. During the period of acclimatization, optimum water quality was maintained with temperature ranges from 26 to 28 °C, dissolved oxygen 5.7 ± 0.7 mg L^−1^, pH 7.4–7.7 and 12:12 h of light: dark photoperiod.

### Experimental protocol and sampling

Before the experimental setup, fishes were starved for 24 h to decrease the ammonia load in transport water. Before transportation, the non-treated fishes were sampled and the water quality parameters were analyzed. The fishes were packed in polythene packets (45 × 30 cm) containing 1 L water and filled with medical-grade oxygen. The total weight of fish in each packet was kept as 100 ± 3.5 g L^−1^ of water (Mean weight ± SD). These packets were divided into four treatments (T_1_—0.1%, T_2_—0.2%, T_3_—0.3% and T_4_- 0.4% D-glucose/L of water) in triplicate along with the control (T_0_). Analytical grade D-glucose was procured from Sigma-Aldrich, India (CAS # 50-99-7). The plastic bags were then sealed, placed in Styrofoam boxes, and subjected to simulated transportation for 12 h in a mechanical shaking platform (mean speed 110 rpm). Serum and tissues (liver, gill, muscle) sampling were conducted at various intervals. Initially, the sampling was done before the transportation (0 h) and immediately after 12 h of transportation (1st day). After that, the remaining live fishes of each treatment and control were stocked in experimental tanks of 35 L each in triplicate**.** Further**,** In order to stop the additional energy expenditure in energy-depleted transported fishes, no feeding was given up to 36th h (2nd day) sampling. After 2nd day sampling, feeding was provided until 168th h (7th day) in all experimental groups to aid the stressed fish in recovery. Siphoning was also done daily from 3^rd^ to 7th day to remove the excess faecal matter, and round the clock aeration was maintained in each experimental tank. Further, the cumulative survivability of fish at each treatment and control was monitored from pre-transportation to 7 days’ post-transportation.

### Water quality parameters

Water quality parameters such as pH value was recorded by digital pH meter (LABINDIA) and the Total ammonia–nitrogen was measured in the pre and post-experimental period according to standard methods of APHA^45^.

### Biochemical biomarkers in serum

Carp specific cortisol ELISA monoclonal kit Cayman Chemicals (USA) was used for determining the serum cortisol hormone level. Serum Glutamate Pyruvate Transaminase Enzyme (SGPT) and serum triglyceride level were analyzed by CK-NAC kits (NAC activated method, kinetic, Erba Mannheim, Transasia Bio-medicals, Daman, India). Serum creatinine level and glucose level were also assayed using CK-NAC kit.

### Tissue biomarkers

Superoxide dismutase (SOD: EC 1.15.1.1) was assayed according to the method described by Mishra and Fridovich^46^ based on the oxidation of epinephrine-adrenochrome transition by the enzyme. The reaction mixture includes 50 µL of the sample and 1.5 mL 0 1 M carbonate- bicarbonate buffer containing 57 mg  dL^−1^ EDTA (pH 10.2) and 0.5 mL epinephrine (3 mM) and optical density was recorded at 480 nm for 3 min in a Shimadzu—UV spectrophotometer. SOD activity was expressed as unit activity (amount of enzyme required to give 50% inhibition of epinephrine auto oxidation).

Catalase (CAT: EC 1.11.1.6) activity was assayed by the standard protocol of Takahara et al.^47^. Enzyme source was mixed to 2.45 ml phosphate buffer (50 mM, pH 7.0) and 1.0 ml of H_2_O_2_ solution was added to it. The decrease in the absorbance was then measured at 240 nm at intervals for 3 min. CAT activity was expressed as nano-moles H_2_O_2_ decomposed/ min/ mg protein.

Metabolizing biomarkers such as LDH (EC 1.1.1.27) and MDH (EC 1.1.1.37) activities were evaluated in liver and muscle tissue by using the method of Wrobleiuski and Ladeu^48^ and Ochoa^49^. The reaction mixtures for both LDH and MDH were prepared using 2.7 mL of 0.1 M phosphate buffer (pH 7.5), 0.1 mL of NADH solution (2 mg NADH dissolved in 1 mL of phosphate buffer solution), 0.1 mL of tissue homogenate and substrate (1 mL of 0.1 mM sodium pyruvate for LDH and 0.1 mL of 0.1 mM oxaloacetate solution for MDH). The OD was recorded at 340 nm at 30 s interval. LDH and MDH activity was expressed as units  mg^−1^ protein min^−1^ at 25 °C where 1 unit was equal to A 0.01 OD min^−1^.

The glucose-6-phosphatase (G-6Pase; EC 3.1.3.9) activity was analyzed in liver tissue by the standard method of Marjorie^50^. During the estimation, 0.3 mL of malate buffer (pH 6.5), 0.1 mL of 0.1 M glucose 6-phosphate solutions and 0.1 mL of tissue homogenate and was mixed and incubated for 15 min at 37 °C. After that 10% TCA solution was added and OD was measured at 600 nm. G-6Pase activity was expressed as nanomoles of inorganic phosphorous released/ minute/ mg protein.

### Isolation of RNA and real-time quantitative PCR

Isolation of Total RNA was achieved by the TRIZOL reagent (Invitrogen Life Technologies, USA) using the standard protocol of Chomczyński and Sacchi^51^. After that, the elimination of genomic DNA from the RNA sample was achieved by treating the sample with DNase (Thermo Scientific, USA) at 37 °C for 40 min following the manufacturer's methods. After that, total concentration and purity of the DNase treated sample were measured by Nanodrop spectrophotometer, USA at 260 and 280 nm. Further, the quality of the mRNA was checked by running it on a 2% agarose gel. cDNA synthesis was then done by the Verso cDNA Synthesis kit (ThermoFisher scientific, USA) following the manufacturer's protocol. The HSP 70 primers are designed based on a homologous region in the HSP 70 family (Accession no: KF737867.1). Thus, the expression data can represent both constitutive and inducible forms. The details about the primers used are represented in Table 3. The mRNA transcript levels of hepatic HSP 70 and house-keeping gene (β-actin) was done by real-time quantitative PCR (qPCR) technique by using the Light Cycler Real-time PCR detection system (Roche, USA) with SYBR Green as a florescent cyanide dye (Thermo Fisher Scientific, USA). From the Light Cycler Real-time PCR (Roche, USA) software, the threshold cycle (Ct) values were obtained, then each Ct value of target gene was normalized with the corresponding Ct value of β-actin and the value was represented in an n-fold difference relative to the defined control. The Ct (2 − ΔCt) value method^52^ was used for determining the relative expression level of the target gene.

**Table 3:** Details of primers used for real time gene expression.

β‑Actin F	CACTGCTGCTTCCTCCTCCTC	EU184877.1
β‑Actin R	GATACCGCAAGACTCCATACC	
HSP-70 F	GTGTTTGATGCCAAGAGGCTG	KF737867.1
HSP-70 R	CTGCATTTGTCACCTTCTGCC	

### Integrated biomarker response (IBR)

Integrated biomarker responses (IBRs) were estimated for different biomarkers in various organs (liver, gill, muscle) and serum at different glucose levels, and respective star plots were depicted to evaluate the multi-biomarker response in *L. rohita*. The analysis was performed according to the methodology proposed by Beliaeff and Burgeot^53^ with modifications by Guerlet et al.^54^. Star plots were depicted to represent the scores (S) of all biomarkers measured in a given treatment and tissue, as well as to calculate IBRs according to the following formulas:

$$ {\text{Ai }} = {\text{ Si}}/{\text{2 sin}}\beta \, \left( {{\text{Si cos}}\beta \, + {\text{ Si}} + {\text{1 sin}}\beta } \right) $$

where β = Arc tan (Si + 1 sinα/ Si − Si + 1 cosα) and α = 2π/ n, Sn + 1 = S1.

where Ai is the area connecting the two scores (S), Si and Si + 1 are two consecutive clockwise scores (radius coordinates) of a given star plot, and *n *is the number of biomarkers used for calculations. The IBR index for each treatment for different tissue (gills, liver, muscle) and serum was then standardized to calculate the mean value of each biomarker.

### Statistical analysis

The statistical package SPSS 16.0 (IBM statistic 16.0) was used to analyze the differences in mean values of the parameters estimated of the control and treatments (four different concentrations of D-Glucose) followed by Duncan’s multiple range test. Results are expressed in mean ± standard error.

### Guidelines

This is to state that on live vertebrates (Fish), confirm that all experiments were carried out in compliance with the ARRIVE guidelines and were performed in accordance with relevant guidelines and regulations, as per Government of India followed in the Institute, ICAR-Central Institute of Fisheries Education (University under section 3 of the University Grants Commission Act and ISO 9001:2008 certified), Mumbai, India.

### Ethics statement

This is to state that all methods and all the experimental protocols were carried out in accordance with relevant guidelines and regulations and all the experimental protocols were approved by ICAR-Central Institute of Fisheries Education (Deemed University), Mumbai, India.

## Supplementary Information

Supplementary Information.
