# Supplementary material for: An Integrated biomarker approach for explaining the potency of exogenous glucose on transportation induced stress in Labeo rohita fingerlings
Source: Sci Rep. 2021 Mar 11;11:5713. doi: 10.1038/s41598-021-85311-5 (PMC7970946; doi:10.1038/s41598-021-85311-5)
Supplement: Supplementary file 1 — Supplementary Information. [file 41598_2021_85311_MOESM1_ESM.docx]

**Title of Manuscript: An Integrated Biomarker Approach for Explaining the Potency of Exogenous Glucose on Transportation Induced Stress in *Labeo rohita* Fingerlings**

**Author Names:** Abhilipsa Biswal^1^, Prem Prakash Srivastava^1*^, Gopal Krishna^1^, Tapas Paul^1^, Prasenjit Pal^2^, Subodh Gupta^1^, Tincy Varghese^1^ and Manish Jayant^1^

*^1^ICAR- Central Institute of Fisheries Education, Mumbai- 400 061, India*

*^2^College of Fisheries, Central Agricultural University, Lembucherra, Tripura, 799210, India*

**Corresponding Author:* P.P. Srivastava ([ppsrivastava@cife.edu.in](mailto:ppsrivastava@cife.edu.in))

| **Parameter** | **Treatment** | **Before Transportation** | **After transportation** |
| --- | --- | --- | --- |
| Total Ammonia  (mg N/L) | T_0_ (Control) | 0.18^a^ ± 0.01 | 6.52^a^ ± 0.18 |
|  | T_1_ (0.1%) |  | 5.85^b^± 0.26 |
|  | T_2_ (0.2%) |  | 4.47^d^ ± 0.24 |
|  | T_3_ (0.3%) |  | 5.31^c^ ± 0.33 |
|  | T_4_ (0.4%) |  | 5.85^b^ ± 0.15 |
| pH | T_0_ (Control) | 6.9^a^ ± 0.02 | 6.2^c^ ± 0.22 |
|  | T_1_ (0.1%) |  | 6.8^b^ ± 0.19 |
|  | T_2_ (0.2%) |  | 7.3^a^ ± 0.31 |
|  | T_3_ (0.3%) |  | 7.5^a^ ± 0.34 |
|  | T_4_ (0.4%) |  | 7.4^a^ ± 0.28 |

**Supplementary table S1:** Total ammonia-nitrogen and pH level in experimental water (before and after transportation) with addition of glucose. Data is represented in mean ± SE, p value ≤ 0.05 and various superscripts represent the significant levels.

| **Parameter** | **Treatments** | **Liver** | | | | **Muscle** | | | |
| --- | --- | --- | --- | --- | --- | --- | --- | --- | --- |
|  |  | **Before Transportation** | **1^st^ Day** | **2^nd^ Day** | **7^th^ Day** | **Before Transportation** | **1^st^ Day** | **2^nd^ Day** | **7^th^ Day** |
| **LDH**  (micromoles/mg protein/min) | T_0_ (Control) | 4.82^a^ ± 0.12 | 8.46^a^ ± 0.19 | 6.81^a^ ± 0.23 | 5.25^a^ ± 0.17 | 8.17^a^ ± 0.41 | 20.11^a^ ± 1.50 | 15.13^a^ ± 0.68 | 9.23^a^ ± 0.24 |
|  | T_1_ (0.1%) |  | 7.52^a^ ± 0.17 | 5.92^b^ ± 0.18 | 5.03^ab^ ± 0.13 |  | 15.29^a^ ± 1.13 | 13.24^a^ ± 1.02 | 8.84^a^ ± 0.11 |
|  | T_2_ (0.2%) |  | 6.11^b^ ± 0.09 | 5.01^c^ ± 0.11 | 4.84^b^ ± 0.08 |  | 11.09^b^ ± 0.86 | 9.62^b^ ± 0.82 | 8.32^b^ ± 0.13 |
|  | T_3_ (0.3%) |  | 6.25^b^ ± 0.09 | 5.22^c^ ± 0.12 | 4.91^ab^ ± 0.05 |  | 12.16^b^ ± 1.05 | 9.39^b^ ± 1.10 | 8.38^b^ ± 0.14 |
|  | T_4_ (0.4%) |  | 6.28^b^ ± 0.11 | 5.67^c^ ± 0.08 | 4.96^ab^ ± 0.07 |  | 12.28^b^ ± 1.16 | 9.68^b^ ± 0.43 | 8.44^b^ ± 0.09 |
| **MDH**  (micromoles/mg protein/min) | T_0_ (Control) | 0.25^a^ ± 0.02 | 0.95^a^ ± 0.04 | 0.67^a^ ± 0.04 | 0.40^a^ ± 0.03 | 1.20^a^ ± 0.08 | 2.50^a^± 0.06 | 2.02^a^ ± 0.04 | 1.38^a^ ± 0.05 |
|  | T_1_ (0.1%) |  | 0.78^b^ ± 0.04 | 0.53^b^ ± 0.04 | 0.36^a^ ± 0.02 |  | 2.12^b^ ± 0.06 | 1.82^b^ ± 0.05 | 1.32^a^ ± 0.06 |
|  | T_2_ (0.2%) |  | 0.59^c^ ± 0.02 | 0.38^c^ ± 0.03 | 0.27^a^ ± 0.04 |  | 1.71^c^ ± 0.04 | 1.49^c^ ± 0.04 | 1.22^a^ ± 0.04 |
|  | T_3_ (0.3%) |  | 0.62^c^ ± 0.03 | 0.40^bc^ ± 0.03 | 0.28^a^ ± 0.06 |  | 1.73^c^ ± 0.04 | 1.52^c^ ± 0.03 | 1.26^a^ ± 0.06 |
|  | T_4_ (0.4%) |  | 0.64^c^ ± 0.02 | 0.42^bc^ ± 0.04 | 0.28^a^ ± 0.05 |  | 1.80^c^ ± 0.03 | 1.50^c^ ± 0.03 | 1.27^a^ ± 0.04 |

**Supplementary table S2:** LDH and MDH activity in liver and muscle tissue of *L. rohita* fingerlings before and after transportation (mixed with glucose in ambient water). Data is represented in mean ± SE, p value ≤ 0.05 and various superscripts represent the significant levels.

| **Parameter** | **Treatments** | **Liver** | | | | **Gill** | | | |
| --- | --- | --- | --- | --- | --- | --- | --- | --- | --- |
|  |  | **Before Transportation** | **1^st^ Day** | **2^nd^ Day** | **7^th^ Day** | **Before Transportation** | **1^st^ Day** | **2^nd^ Day** | **7^th^ Day** |
| **SOD**  (U mg protein^-1^) | T_0_ (Control) | 13.9^a^± 0.23 | 23.6^a^± 1.18 | 17.34^a^± 0.82 | 14.01^a^± 0.91 | 17.6^a^± 0.87 | 38.3^a^± 1.87 | 21.8^b^± 1.19 | 16.9^a^± 0.88 |
|  | T_1_ (0.1%) |  | 20.5^b^± 1.02 | 16.84^a^± 0.55 | 13.61^a^± 0.73 |  | 31.2^b^± 1.75 | 24.3^a^± 1.77 | 16.2^a^± 0.65 |
|  | T_2_ (0.2%) |  | 15.8^d^± 0.67 | 13.25^c^± 0.44 | 10.91^b^± 0.66 |  | 23.3^d^± 1.59 | 19.2^d^± 0.69 | 14.5^c^± 0.53 |
|  | T_3_ (0.3%) |  | 17.4^c^± 0.35 | 15.85^b^± 0.61 | 11.81^b^± 0.54 |  | 25.1^d^± 1.92 | 19.6^d^± 0.83 | 14.3^c^ ± 0.44 |
|  | T_4_ (0.4%) |  | 17.7^c^± 0.46 | 15.70^b^± 0.38 | 13.10^a^± 0.87 |  | 28.8^c^± 1.16 | 20.4^c^± 0.58 | 15.5^b^± 0.38 |
| **CAT**  (mmole mg protein^-1^ min^-1^) | T_0_ (Control) | 0.75^a^± 0.03 | 1.51^a^± 0.09 | 1.23^a^± 0.07 | 0.79^a^± 0.05 | 4.3^a^± 0.28 | 8.48^a^± 0.36 | 5.99^a^± 0.37 | 4.32^a^± 0.32 |
|  | T_1_ (0.1%) |  | 1.34^b^± 0.05 | 1.20^a^ ± 0.08 | 0.74^a^± 0.04 |  | 7.57^b^± 0.28 | 5.62^ab^± 0.41 | 4.19^a^± 0.23 |
|  | T_2_ (0.2%) |  | 1.12^d^± 0.02 | 0.89^c^± 0.03 | 0.70^a^± 0.03 |  | 6.89^c^± 0.49 | 4.30^c^ ± 0.22 | 3.78^b^± 0.26 |
|  | T_3_ (0.3%) |  | 1.06^e^± 0.04 | 0.97^b^± 0.07 | 0.75^a^± 0.06 |  | 6.56^c^± 0.44 | 4.98^c^± 0.31 | 3.89^b^± 0.18 |
|  | T_4_ (0.4%) |  | 1.21^c^± 0.03 | 1.05^b^± 0.06 | 0.73^a^± 0.04 |  | 7.68^b^± 0.32 | 5.21^b^± 0.48 | 4.12^a^± 0.35 |

**Supplementary table S3:** SOD and CAT activity in liver and gill tissue of *L. rohita* fingerlings before and after transportation (mixed with glucose in ambient water). Data is represented in mean ± SE, p value ≤ 0.05 and various superscripts represent the significant levels.

**Supplementary fig. S1:** G-6 phosphatase concentration in liver of *Labeo rohita* fingerlings before and after transportation (mixed with glucose in ambient water). Data is represented in mean ± SE, p value ≤ 0.05 and various superscripts represent the significant levels.
